# An Organogold Compound as Potential Antimicrobial Agent against Drug‐Resistant Bacteria: Initial Mechanistic Insights

**DOI:** 10.1002/cmdc.202100342

**Published:** 2021-07-23

**Authors:** Parichita Chakraborty, Dorenda Oosterhuis, Riccardo Bonsignore, Angela Casini, Peter Olinga, Dirk‐Jan Scheffers

**Affiliations:** ^1^ Department of Molecular Microbiology Groningen Institute for Biomolecular Sciences and Biotechnology University of Groningen 9747 AG Groningen The Netherlands; ^2^ Department of Pharmaceutical Technology and Biopharmacy Groningen Research Institute of Pharmacy University of Groningen 9713AV Groningen The Netherlands; ^3^ Chair of Medicinal and Bioinorganic Chemistry Department of Chemistry Technical University of Munich Lichtenbergstr. 4 85748 Garching b. München Germany

**Keywords:** Antibiotics, gold compounds, organometallic drugs, drug resistant bacteria, mode of action.

## Abstract

The rise of antimicrobial resistance has necessitated novel strategies to efficiently combat pathogenic bacteria. Metal‐based compounds have been proven as a possible alternative to classical organic drugs. Here, we have assessed the antibacterial activity of seven gold complexes of different families. One compound, a cyclometalated Au(III) C^N complex, showed activity against Gram‐positive bacteria, including multi‐drug resistant clinical strains. The mechanism of action of this compound was studied in *Bacillus subtilis*. Overall, the studies point towards a complex mode of antibacterial action, which does not include induction of oxidative stress or cell membrane damage. A number of genes related to metal transport and homeostasis were upregulated upon short treatment of the cells with gold compound. Toxicity tests conducted on precision‐cut mouse tissue slices *ex vivo* revealed that the organogold compound is poorly toxic to mouse liver and kidney tissues, and may thus, be treated as an antibacterial drug candidate.

## Graphical abstract

**Genetic‐level effects**: We report the antibacterial effects of a gold(III) organometallic compound against Gram‐positive bacteria and the evaluation of its toxicity in healthy tissues in a mouse model *ex vivo*. The compound's mechanisms of action, elucidated by various assays in *B. subtilis*, do not include the induction of oxidative stress or bacterial cell membrane damage, but instead involve the alterations of genes related to metal transport and homeostasis, amongst others.

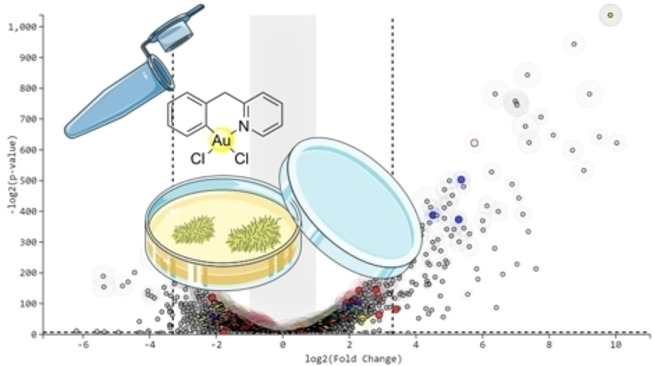

## Introduction

**Figure 1:**
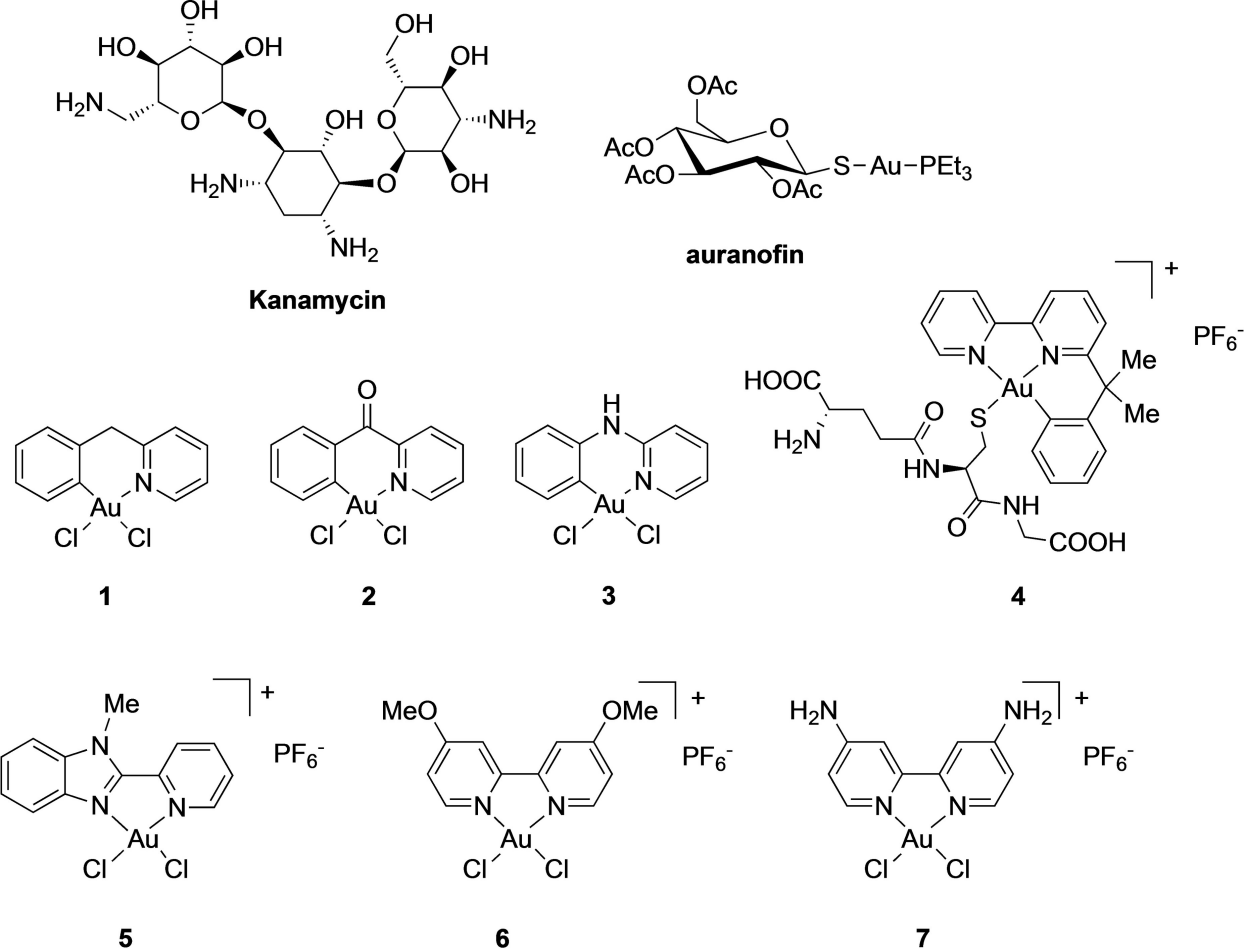
Structures of auranofin and of the Au(III) complexes under investigation, and of the antibiotic Kanamycin.

The interest in using gold‐based substances for therapeutic applications dates back to ancient times and was also an important aspect of alchemy in Medieval Europe and in the Renaissance period, when gold was an essential ingredient of so‐called *aurum vitae* medicines.^[1]^ In recent years, gold(I) compounds have also found clinical relevance as anti‐rheumatic drugs, for example in the form of auranofin ([triethylphosphine(2,3,4,6‐tetra‐O‐acetyl‐*β*‐1‐D‐(thiopyranosato‐S)Au(I)], (Figure 1), and are also currently in clinical development for the treatment of cancer.^[2]^

Interest for antimicrobial gold complexes originated from the work of Robert Koch in the end of 19^th^ century, demonstrating that potassium dicyanidoaurate(I), K[Au(CN)_2_], had activity against *Mycobacterium tuberculosis*.^[3]^ The popularity of gold as an anti‐microbial dwindled with the discovery and subsequent mass‐production of Penicillin, with which modern science and medicine entered into the era of antibiotics.^[4]^ However, the benefits offered by these magic bullets have been substantially rapidly lost following the widespread emergence and dissemination of antibiotic‐resistant bacterial strains.^[5]^ Therefore, in the past years, renewed interest in metallodrugs development as antibacterial agents took place,^[6]^ and different families of Au(I) and Au(III) complexes were investigated for their antibacterial properties, including Au(I) N‐heterocyclic carbenes (NHCs).^[7]^ Early reports of Demir and Özdemir et al. proved the activity of neutral benzimidazolylidene‐based monocarbene Au(I) complexes against Gram‐positive bacteria (*Staphylococcus aureus* and *Enterococcus faecalis*), while having no effect toward Gram‐negative bacteria *in vitro*.^[8]^ In general, most of the Au(I) complexes tested so far display antibacterial effects only on Gram‐positive bacteria, with a few exceptions.^[9]^

Concerning possible mechanisms of action, disruption of the bacterial cell wall and cytosolic membrane by gold complexes in different bacteria strains was postulated, also based on morphological changes of the bacteria's surface and on cytosolic leakage as observed by scanning electron microscope (SEM) measurements.^[10]^ Docking studies suggested that the peptidoglycan layer of the bacterial membrane is destabilized by the gold compounds’ insertion in the lipid bilayer followed by the loss of structural integrity.^[10]^ Overall, the selectivity of certain Au(I) complexes towards Gram‐positive bacteria strains can be explained by the different construction of the cell membrane. While Gram‐positive bacteria have only a single layer membrane, Gram‐negative strains feature an inner and an outer membrane that also includes efflux transporters.^[11]^

Of note, in 2015, Schultz and Wang showed that *M. tuberculosis* is susceptible to auranofin, in addition to a variety of additional pathogenic Gram‐positive bacteria.^[12]^ Moreover, auranofin was efficacious in a murine model of methicillin‐resistant *S. aureus* infection.^[12]^ The study also reported on the potent inhibition of bacterial thioredoxin reductase (TrxR) by auranofin, showing that the compound disrupts the intracellular redox balance, eventually leading to bacterial cell death.[^12^, ^13^] Similar potent inhibition of bacterial TrxR was reported for Au(I) NHC complexes featuring activity in Gram‐positive bacterial strains.^[14]^ It is worth noting that, while in several organisms glutathione (GSH) and GSH reductase (GR) function in parallel with thioredoxin (Trx) and thioredoxin reductase (TrxR) to provide the cell with a source of reducing equivalents,^[15]^ most Gram‐positive strains lack the GR/GSH system,^[16]^ relying more on the Trx/TrxR one to detoxify reactive oxygen species (ROS). Therefore, inhibition of TrxR by gold complexes makes Gram‐positive bacteria more vulnerable to their treatment.

Among the Au(III) complexes that have been tested for antimicrobial potential, early studies in the 90s highlighted Au(III) dithiocarbamates, derived from amino acids, that exhibited a larger activity against *Streptococcus pneumoniae* than the reference antimicrobial agents.^[17]^ Moreover, Parish et al. have carried out a range of biological tests to evaluate [AuCl_2_(damp)] (damp=2‐((dimethylamino)methyl)‐phenyl) and other cyclometalated derivatives for antimicrobial activity against a panel of bacteria, representing microorganisms of clinical importance.^[18]^ The latter compounds showed moderate activities and a certain preference for Gram positive bacterial strains.^[7]^

More recently, Arca and co‐workers reported on the promising antimicrobial activity of cyclometalated Au(III) complexes against *Staphylococcus* strains, although in‐depth mechanistic studies were not conducted.^[19]^ The clinical relevance of some bis(pyrrolide‐imine) Au(III) macrocycles as topoisomerase 1A inhibitors was also demonstrated by their antibacterial effects in a panel of diverse *M. tuberculosis* and *M. abscessus* clinical isolates.^[20]^ However, so far only limited attention has been paid to the application of Au(III) compounds as potential antimicrobial agents and to the elucidation of their modes of action.

In this context, we decided to expand the investigation on Au(III) complexes and selected seven representative molecules – featuring bidentate N^N ligands, cyclometalated bidentate C^N and terdentate C^N^N scaffolds, respectively – for antibacterial screening (compounds **1**–**7**, Figure 1). The selection was initially performed based on previous results on the antiproliferative activities of the compounds in human cancer cells.^[21]^ Specifically, the compounds were chosen based on their scarce cytotoxic activity, in order to exclude side effects in mammalian cells.

Of note, compounds **1**–**4** were selected amongst a family of organometallic derivatives endowed with increased stability in aqueous environment relative to the Au(III) coordination complexes **5**–**7**. For example, density functional theory (DFT) calculations have shown that compound **1** is less prone to ligand exchange reactions with nucleophiles, such as thiol groups of cysteine residues, than the cationic coordination complex **5**.^[22]^ In general, a number of studies demonstrated that Au(III) complexes with bidentate N^N ligands, as in **5**–**7**, can easily hydrolyse the chlorido ligands and even undergo the rapid loss of the chelating ligand upon reaction with different protein binding sites.^[23]^ Moreover, experimental studies and calculations highlighted mixed chloro‐hydroxo species to be the dominant ones for the reaction with endogenous targets.[^21d^, ^24^] Despite this reduced stability, we have included the coordination Au(III) complexes in our screening since some of them have been reported to efficiently inhibit bacterial enzymes *in vitro*.^[25]^

**Scheme 1:**
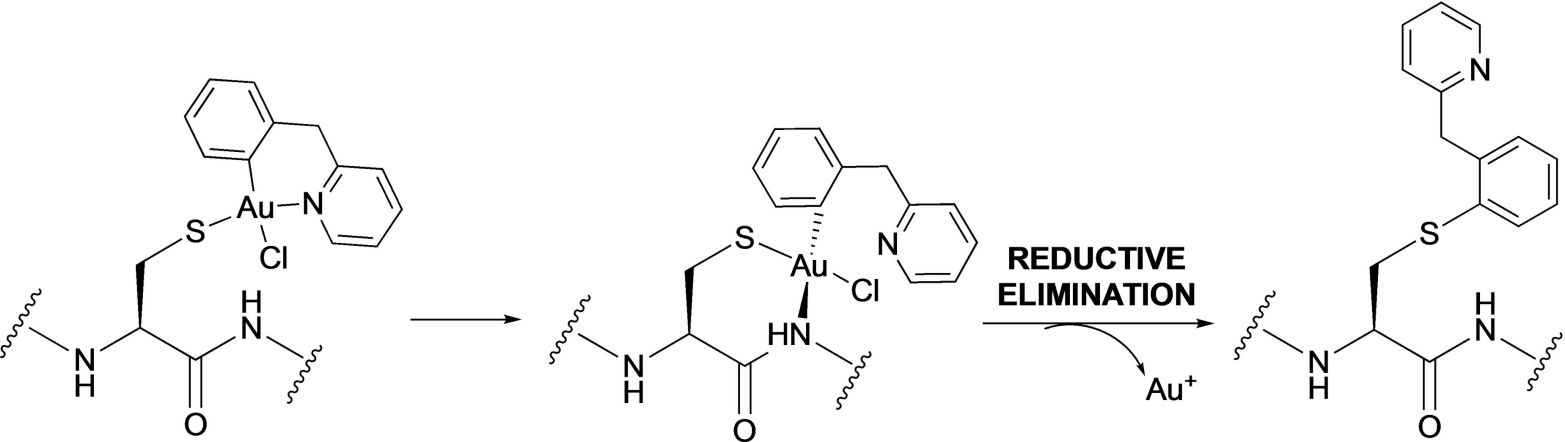
Reaction of cyclometalated Au(III) complexes **1**–**3** with cysteine residues.

Interestingly, compounds **1**–**3** also feature a peculiar reactivity with cysteine residues.[^23^, ^26^] Specifically, it has been demonstrated that, following AuC^N‐Cys adduct formation, the reaction of complexes **1**–**3** with peptides proceeds towards cysteine arylation (Scheme 1).

Combined mass spectrometry and DFT calculations showed that formation of the covalent C^N‐peptide adduct is templated by the Au(III) centre facilitating the C−S cross‐coupling reaction via reductive elimination.^[27]^ Instead, complexes **4**–**7** are known to bind amino acid residues only establishing Au‐based adducts following exchange of the chlorido and eventually of the bidentate ligands.^[28]^

## Results and Discussion

### Antimicrobial activity of the gold compounds

Initially, Minimum Inhibitory Concentrations (MICs) for the seven Au(III) compounds were determined by broth microdilution (see Experimental Section for details) against *E. coli* MG1655 (Gram‐negative bacterium) and *B. subtilis* 168 (Gram‐positive bacterium). While all the compounds were inactive towards *E. coli*, complexes **1** and **4** showed an inhibitory effect on the growth of *B. subtilis* colonies (Table 1, Table S1), with MIC values only slightly higher than Kanamycin,^[29]^ a potent antibiotic used as positive control (12.5 μM and 6.25 μM, respectively for **1** and **4**, vs 3.12 μM). The difference in activity between compound **1** and the two C^N analogues **2** and **3** has also been observed in previously reported antiprotozoal screening,^[30]^ and may be due to different reasons. For example, complex **2** is extremely reactive with thiols which may be present in the extracellular environment, leading to inactivation prior to cell uptake. Further differences in cell uptake mechanisms and accumulation amongst the compounds may also play a role and should be further investigated.

**Table 1:** Summary of the MIC values (μM) of **1** and **4** in *E. coli, B. subtilis, S. aureus* and *E. faecium* strains. MIC values are reported as a range between the highest concentration of compound allowing bacterial growth and lowest concentration inhibiting bacterial growth after 24 h.

Compound	Gram negative	Gram positive				
	*E. coli*	*B. subtilis*	*S. aureus RN4220*	*Methicillin‐resistant S. aureus (MRSA)*	*E. faecium Van A (VRE)*	*E. faecium*
Kanamycin	12.5–25	1.56–3.12	50–100	50–100	>100	>100
1	50–100	6.25–12.5 (<12.5^[a]^)	3.12–6.25	6.25–12.5	25–50	25–50
4	>100	3.12–6.25 (<12.5^[a]^)	3.12–6.25	12.5–25	50–100	>100

[a] MBC values are reported in brackets as lower than the minimum concentration that, after incubation of cells with drugs for 24 h, inhibited outgrowth of colonies on compound‐free plates after 24 h. Values shown here are the mean values performed for 2 biological replicates with 2 technical replicates each in case of *B. subtilis* and *E. coli*, 1 technical replicate each for *E. faecium* and 2 biological replicates with 3 technical replicates each in *S. aureus*.

To gain more insights into the antibacterial activity of the two effective Au(III) complexes **1** and **4**, we calculated the Minimum Bactericidal Concentration (MBC) in *B. subtilis* by spotting samples from the MIC plate on LB agar plates without antibiotics (Table 1). Surprisingly the two organometallic complexes showed a different behaviour. Thus, the cells from the 12.5 μM well‐spot of **1** did not show any colony growth, even after 48 hours of plating, suggesting a bactericidal effect of the compound (Table 1). Conversely, **4**‐treated cells developed colonies from the 6.25 μM and 12.5 μM‐treatment well‐spots after 24 and 48 hours of plating, respectively. The latter result suggests that **4** behaves as a bacteriostatic agent.

Intrigued by the two compounds antibacterial activity towards *B. subtilis*, we further screened **1** and **4** in a panel of four clinically relevant Gram‐positive bacterial strains: *Enterococcus faecium* P13, vancomycin resistant *E. faecium* Van A (VRE), *S. aureus* RN4220, and Methicillin Resistant *S. aureus* (MRSA) D22 (Table 1). The latter strain is resistant to methicillin and β‐lactam antibiotics and, depending on the clinical isolate, also to several other antibiotics such as erythromycin, gentamycin, ciprofloxacin and others.^[31]^ Both compounds were active only towards the *S. aureus* strains, but with compound **4** slightly less active in the MRSA strain, with MIC values between 12.5–25 μM (Table 1).

Overall, the obtained results highlight that the two selected organometallic complexes display antibacterial activity towards Gram‐positive bacteria, also including clinically relevant antibiotic‐resistant strains. Further assays were therefore, carried out in *B. subtilis* to determine the mechanism of inhibition of bacterial growth.

### Real‐time killing dynamics

A real‐time killing dynamics assay was performed to determine the speed of antibiotic action. *B. subtilis* cells at OD_600_ 0.2 were incubated with compounds **1** and **4**, and the OD_600_ was measured in real‐time to determine the point at which the antimicrobial activity of the compound affected the growth curve. In this assay, Kanamycin showed significant activity only after 30 min of addition of the drug at a concentration of 4 times the MIC (Figure S1A). The killing dynamics curve of nisin, another fast‐acting antibiotic used as a control, showed fast activity at half the MIC (Figure S1B), and a continuous decrease in OD_600_, due to the fact that nisin kills the cells immediately by inducing formation of large pores in the cell membrane.^[32]^

**Figure 2:**
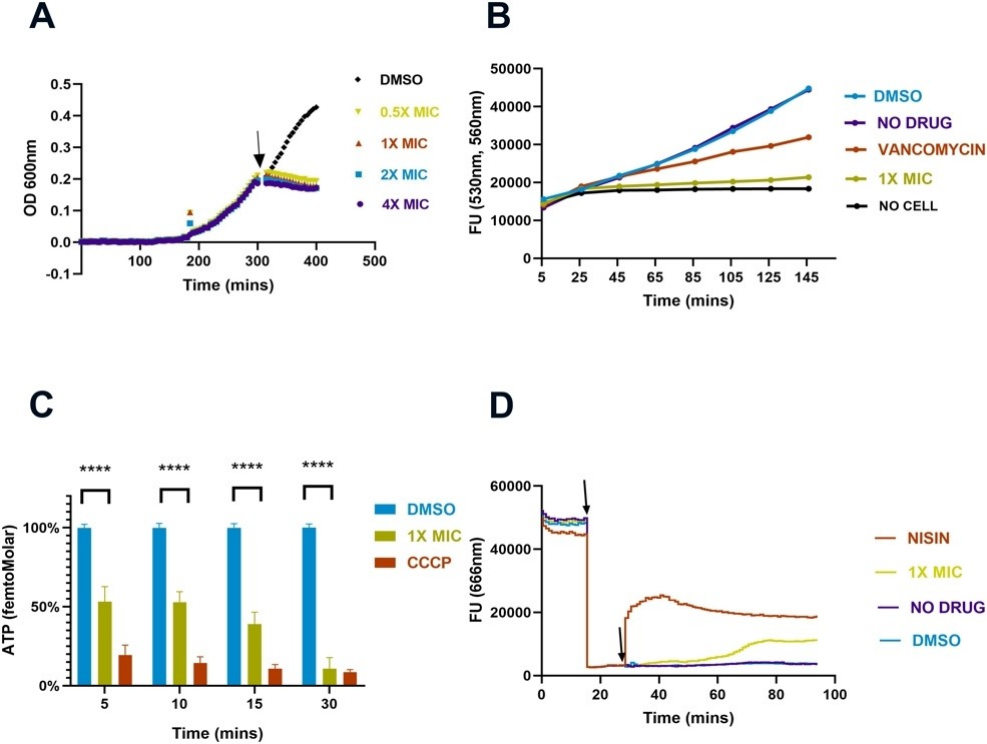
**A**) Killing dynamics for **1** in *B. subtilis* at different concentrations. The y‐axis shows the absorbance at 600 nm. Cells were allowed to grow till OD_600_ 0.2. The arrow indicates the time‐point of drug entry, and the time of action is estimated to be 30 min. DMSO‐1.25 %. The experiment was performed with two different biological replicates with two technical replicates each for each condition. The curve plots the median values for each time point for one of the biological replicates. **B**) REMA assay to determine NADH levels. The y‐axis shows the fluorescence intensity observed at 560 nm (emission) following excitation at 530 nm vs time (x‐axis). The first reading was taken after 6 min, followed by a reading every 20 min. DMSO‐1.25 %. Vancomycin‐1 μg/mL. The experiment was performed with 3 biological replicates and 3 technical replicates each. The graph plots the median values at each time point of one biological replicates. **C**) ATP luciferase assay. The y‐axis shows the ATP concentration vs time (x‐axis). DMSO‐1.25 %. CCCP‐20 μg/mL; **** *p*<0.0001 (ordinary one‐way Anova performed on the percentage data). The experiment was performed with 3 biological replicates and 2 technical replicates each. The graph shows the mean with SD of each condition as percentage of the DMSO sample. **D**) Membrane potential assay with DiSC_3_ showing the depolarization effect of **1**. The first arrow indicates point of cell addition and second arrow indicates point of compound/controls addition. The y‐axis reports the fluorescence intensity observed at 666 nm (emission) following excitation at 643 nm vs time (x‐axis). Nisin‐6 μg/mL. The experiment was performed for 2 biological replicates and 3 technical replicates each. The graph plots the median value at different time point of 1 biological replicate.

Concerning the gold complexes, the assay showed that **1** is a fast‐acting drug, inducing a reduction in the growth rate of the treated cells compared to untreated ones within five min of incubation at half the MIC (Figure 2A). The killing dynamics curve of **4** shows that the compound is slow acting compared to **1** (Figure S1C), with a decrease in growth rate appearing after about 30 min following addition of the compound and only at concentrations around the MIC value and above. These results are in line with the observation from the MBC assay that **1** is bactericidal rather than bacteriostatic, impacting bacterial growth much faster than **4** and Kanamycin. However, when compared to nisin, there is only a gradual reduction in OD_600_ over time with the Au(III) compounds, indicating that the cells do not immediately lyse. These results led us to focus the rest of the study exclusively on **1**.

Since OD_600_ does not directly report on the number of cells killed, the number of viable cells in a *B. subtilis* culture treated with **1** (1×MIC) was determined at various time points, by plating diluted cultures and counting the number of colony forming units (cfu's). After 1.5 h incubation with the compound, the number of viable cells in the culture had dropped 10^7^‐fold. However, over time the number of cells in the culture increased again, indicative of growth in the culture upon longer exposure (Figure S2). The control culture stops increasing in total cells earlier as the growth medium gets exhausted. Two possible explanations for the growth of cells after prolonged metallodrug incubation can be given: either resistant cells appear that start growing, or not all cells in the culture are killed and over time **1** loses activity allowing the remaining cells to resume growth.

To test whether **1** loses activity over time, we preincubated the compound in LB medium for 24 hours at 30 °C, and subsequently determined the activity of the compound using a MIC assay. The MIC of the compound went up by a factor of 2, as the compound failed to kill the cells at 12.5 μM but could prevent bacterial growth at 25 μM. (Figure S3). The most likely explanation for this loss of activity is that **1** is undergoing speciation (e. g. reduction, decomposition) over time, although a fraction of **1** remains still active in the medium. Determining the exact cause of activity loss is beyond the scope of this study.

In summary, **1** immediately acts on bacteria, stopping cell growth and reducing the number of viable cells in culture by a factor of 10^7^, but loses antibacterial activity over time. We note that this activity loss may be beneficial when **1** is used as an antibacterial – this would reduce the risk of resistance development in environmental settings such as hospital wastewater.^[33]^

### Effect on NADH levels

To shed light onto the mechanism of action of the compound, the effect of **1** on the bacterial cells’ respiratory activity was studied by measuring the NADH level in *B. subtilis*. Specifically, in the applied assay, NADH reduces resazurin to fluorescent resorufin, and the detection of fluorescence over time can be used as a measure of the cell's respiratory activity.^[20]^ Other antibiotics that do not affect respiration were used as controls to distinguish between an overall reduction of respiratory activity because of growth arrest or cell death, and a specific effect on respiration. As shown in Figures 2B and S4 A, **1**‐treated cells (1×MIC) have low levels of NADH, close to the no‐cell control, whereas treatment with four different antibiotics (vancomycin, ciprofloxacin, rifampicin, tetracycline) did not greatly block cellular respiration (Figure S4A). The effect of **1** on the cellular NADH level increased with increasing concentrations of the compound, with effects already starting at 0.1×MIC. (Figure S4B). This result suggests that the respiratory pathway is a possible target for the gold compound.

### Effect on ATP level

A decrease in respiratory activity is expected to result in a decrease in the ATP level in the cell. Therefore, the ATP levels of *B. subtilis* cells treated with **1** were measured, and the results showed a significant reduction in the total ATP level of the cell, already 5 min after addition of the drug compared to DMSO‐treated cells (Figure 2C). The ATP depletion was maximal after 30 min of drug treatment. As a control, CCCP, a protonophore which blocks ATP generation, was used. CCCP caused an immediate drop in cellular ATP levels. The depletion of ATP with **1** was slower compared to the CCCP control, indicating that ATP synthesis is not directly targeted by the gold compound. Overall, this result agrees with the NADH assay, showing a decrease in the overall energy status of the cell.

### Effect on membrane potential

A decrease in intracellular energy levels could be caused by a drop in membrane potential, which is linked to energy generation. Therefore, the effect of **1** on the membrane potential of *B. subtilis* was studied using the dye DiSC_3_(5), which inserts into negatively charged membranes with concomitant quenching of fluorescence. Membrane depolarization leads to a release of the dye from the membrane and an increase of fluorescent signal, as seen in the positive control with the pore‐forming antibacterial peptide nisin (Figure 2D). **1**‐treated cells do not show an immediate increase in fluorescent signal, and fluorescence only gradually increases after ca. 40 min of incubation. Since **1** is a fast‐acting compound, the effect of depolarization would be expected to be fast. Instead, the increase in fluorescent signal seen after ca. 40 min incubation is likely the result of dye leakage from the membranes of dead cells. Thus, **1** does not seem to act via depolarization of the bacterial membrane.

### Effect on membrane permeability

**Figure 3:**
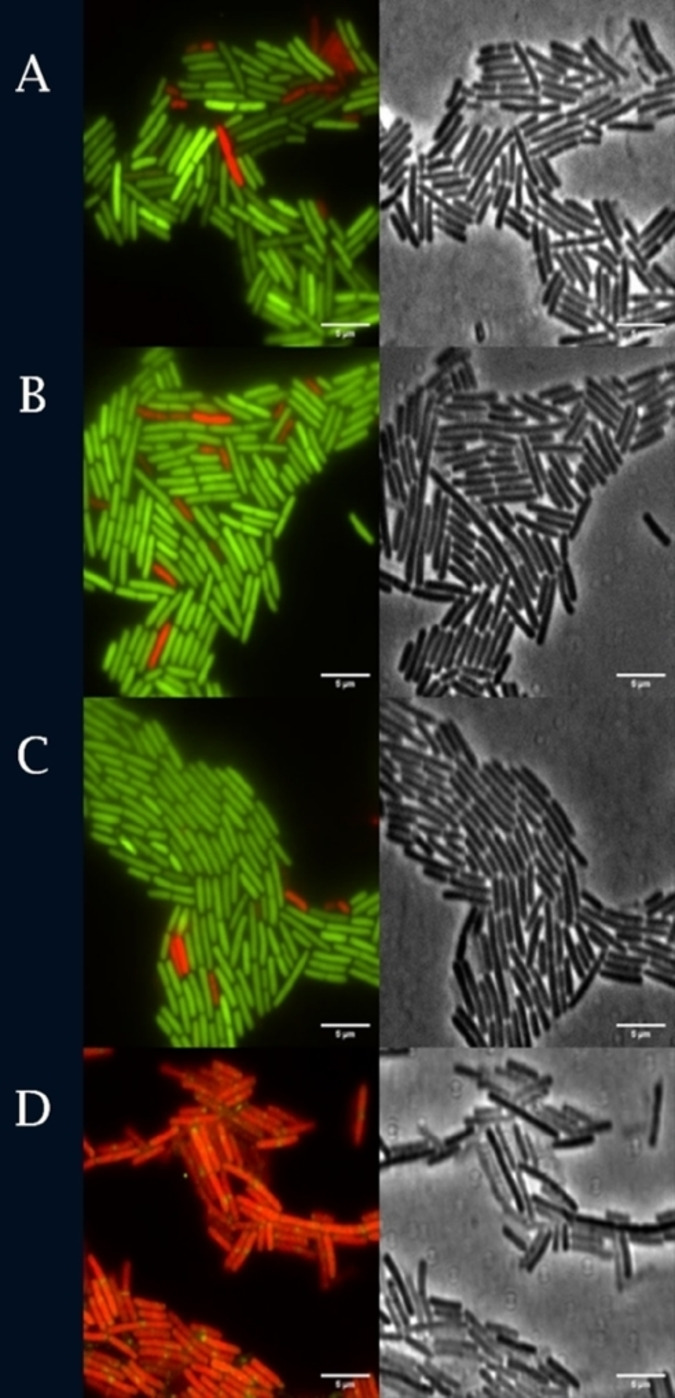
Membrane permeability assay with SYTO9 and PI. **A**) Compound **1** (1×MIC), **B**) DMSO‐1.25 %, **C**) control, **D**) Nisin‐6 μg/mL. Scale bar 5 μm. The experiment was performed for 3 biological replicates with 2 technical replicates in each case. Incubation time was 15 min for samples **A**, **B** and **C** and 10 min for treatment **D**.

To further study the effect of the compound on the membrane integrity, cells were labelled with two DNA stains: SYTO9 and propidium iodide (PI), and inspected by microscopy. SYTO9 is a membrane‐permeable dye and can stain all cells, while PI, being membrane impermeable, can only enter a cell with permeabilized cell membranes. This assay is commercialised as the ‘BacLight Live/Dead assay’, but in earlier work we have shown that cells identified as ‘dead’ in the assay are either dead or have temporarily damaged membranes that can recover.^[34]^ Microscopic inspection of labelled cells revealed that compound **1** did not have any significant effect on membrane permeability (Figure 3A), as the percentage of cells stained with PI is 5.67±2.17 % after treatment with **1**, which is similar to the percentage of stained cells for the no‐drug and DMSO controls (3.73±2.77 % and 4.89±0.99 % respectively). Nisin treatment, on the other hand, resulted in complete cell lysis. The obtained results suggest that compound **1** does not form pores in the bacterial cell membrane. This result also agrees with the result from membrane potential assay presented above, as pores in the cell membrane would be expected to result in a collapse of the membrane potential.

### RNA sequencing

**Figure 4:**
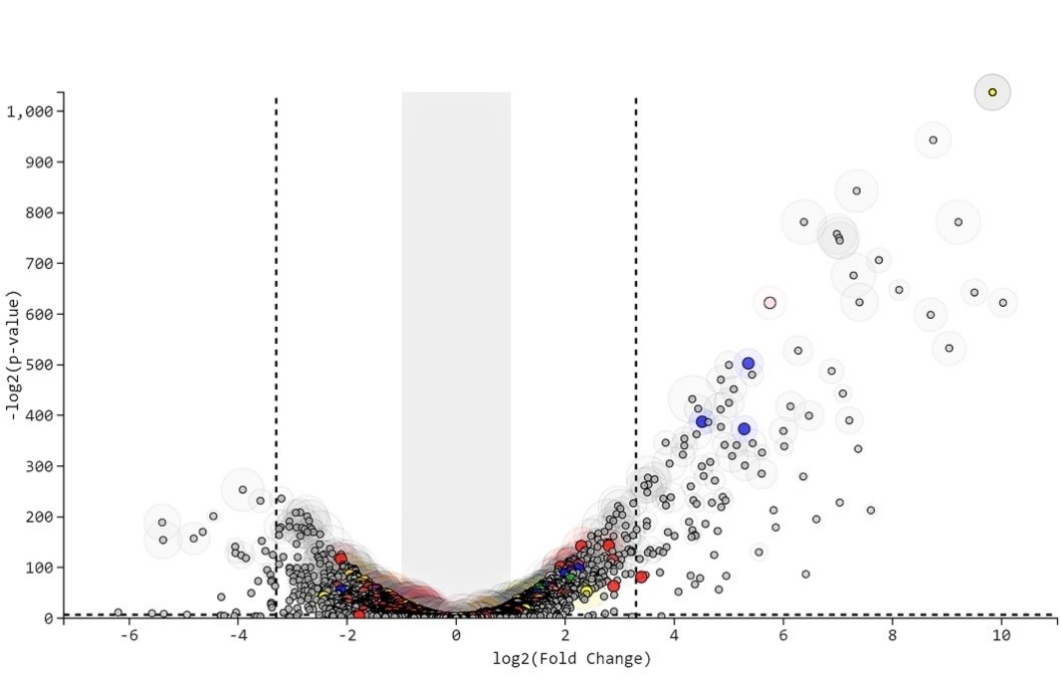
Volcano plot generated using T‐Rex pipeline of *B. subtilis* samples treated with compound **1** (0.2×MIC) for 45 min. Colours represent specific classes of genes. Red‐ Purine/pyrimidine metabolism, Green‐ Drug metabolism, Brown‐ peptidoglycan biosynthesis, Blue‐ Metal metabolism, Yellow‐ Pentose phosphate pathway, Pink‐ Glycolysis/Gluconeogenesis, Purple‐ Citrate cycle, Orange‐ Oxidative phosphorylation. The grey shaded area represents genes that do not show significant difference in their expression pattern. The area within the dotted lines shows the top hits with two‐ to five‐fold changes in expression level. The genes on either side of the dotted line show high fold change genes with greater than equal to five‐fold changes in expression level. The p value above the dotted horizontal line parallel to the x axis is ≤0.01.

To further assess the cellular response to the gold compound's treatment, total RNA sequencing of *B. subtilis* cells treated with **1** was conducted. To detect effects of **1** without killing the cells, RNA was isolated and sequenced from cells treated with non‐cytotoxic concentrations of **1** (0.2×MIC) for 45 and 60 min, respectively. Raw data analysis showed that 149 genes were upregulated and 99 genes were downregulated five‐fold or more after 45 min. The data were further analysed using the T‐Rex pipeline.^[35]^ The volcano plot in Figure 4 shows the differential expression patterns of the genes in the 45 min treated sample. Of the 149 upregulated genes, the top 20 significantly upregulated in the volcano plot, by analysis using the T‐Rex pipeline included: metal transporters like *copA, cadA, czcD*, MFS transporter (*ybcL*), glyceraldehyde‐3‐phosphate dehydrogenase (*gapB*), genes responsible for arsenic metabolism (*arsB, arsC, arsR*), oxidoreductases (*ykvO, ycnD* [FMN reductase], *ydgI* [NADPH nitroreductase]), proteases like *clpE*, transcriptional regulators of MarR family (*ydgJ*), *sat* (sulfate adenylyl transferase), *yxeM* (amino acid binding protein) and several hypothetical genes (*yrkF* [putative sulfur carrier protein], *yrkE* [similar to sulfur transferase], *yrkH, yqcK* [similar to C‐As lyase]). The downregulated genes also belong to different classes, including genes involved in cell wall formation (*manA*), genes involved in contact dependent growth inhibition (*wapA*) and various other ABC transporter genes.

After 60 min incubation with **1**, 176 genes were upregulated and 78 genes were downregulated five‐fold or more from raw data analysis (data not shown). In addition to the genes mentioned for the 45 min treatment, cysP (sulfate permease) is also upregulated and is amongst the top 20 of the volcano plot analysis. Of note, *ykvO*, which is similar to glucose‐1‐dehydrogenase, seems to be the most significantly upregulated gene in both the 45 minute and 60 minute sample.

The transcriptional activators for all of these genes (*sigA* and *sigF*) show down‐regulation in our experimental conditions, but not to a significant level.

We further analysed the interacting gene networks for the upregulated genes using Subtiwiki,^[36]^ an integrated database for *B. subtilis* comprising information on genes, proteins and including regulatory, metabolic and protein‐protein network annotations. An interesting result concerns the upregulation of *cadA*, which is a cadmium transporting ATPase and *czcD*, which is a cation exchanger mainly known to exchange cadmium or zinc in exchange for H^+^ and K^+^. *czcO*, which is similar to Flavin containing mono oxygenase and facilitates cation exchange via *czcD* is also upregulated. Other metal transporters like copper (*copA, copZ*) and sulphate (*yrkF, cysP*) transporters are also upregulated. *arsB, arsC, arsR* and *yqcK*, which are genes involved in arsenic metabolism are also upregulated in our test samples. The protease *clpE* interacts with a number of genes, of which only *clpP, clpC* and *mcsB* show significant upregulation (within top 100 hits in the volcano plot). It is interesting to note that *gapB*, a gene involved in the gluconeogenesis pathway, is the only glucose metabolism gene that is upregulated.

Comparison with a study on condition dependent transcriptome change in *B. subtilis* shows that all the upregulated genes from our study, except two (*gapB* and *sat*), are also upregulated upon treatment with diamide, an agent known to induce disulphide stress.^[37]^ In 2002, another study appeared on the global characterization of genes in *B. subtilis* related to disulfide stress, showing that genes responsible for oxidative stress (under the repression of PerR) and heat shock genes (repression of CtsR) were highly induced.^[38]^ However, none of the oxidative stress genes repressed by PerR were upregulated in our study. Among the heat shock genes repressed by CtsR, only *clpP, clpC, clpE* and *mcsB* were significantly upregulated by the treatment with gold compound. Thus, although we do see some similarity between the genes upregulated in our study and those upregulated by disulfide stress, the overall effect of **1** on the transcriptome is not fully comparable.

Looking at the effect of the compound on the expression levels of other eight different classes of genes, namely purine/pyrimidine metabolism, peptidoglycan biosynthesis, metal metabolism, pentose phosphate pathway, glycolysis/gluconeogenesis, citrate cycle, oxidative phosphorylation and drug metabolism, only genes belonging to the class of ‘metal metabolism’ showed a clear difference in expression profile between the control and treated samples (Figure S5). Significant amongst the latter are *cysH, cysC* and *sat*, showing 20, 36 and 37‐fold upregulation, respectively. The other upregulated genes *cysI, cysE, cysJ, yxjG* were not significantly affected in the volcano plot. All these genes are involved in sulfur metabolism. There are however, three genes (*nasC, nasA* and *nasB*) which are involved with nitrate metabolism and transfer that are downregulated in the treated samples.

A possible explanation for the difference in significance in the volcano plot vs direct expression profile is the weight associated to the fold change difference in the volcano plot. Genes like *ykvO, yrkE* and others show almost 1000× upregulation, which could cause genes with lower fold change difference to appear as not significantly affected in the volcano plot analysis.

Direct expression analysis also revealed downregulation of all the genes involved in ATP synthesis (*atpF, atpE, atpH, atpC, atpD, atpB, atpG, atpA, atpI*), up to almost 10‐fold for *atpF*. This downregulation was not scored as significant in the volcano plot. In general, this analysis shows that **1** kills the bacterial cells by causing general cellular stress. The upregulation of genes encoding metal transporters can be explained by the need of the bacterial cells to react to the toxic gold ions, and the upregulation of proteases such as *clpE* to protein misfolding, but clearly the overall mode of action of the compound is far more complex. For example, the upregulation of *ykvO* and *gapB*, genes involved in the maintenance the NADH/NAD^+^ balance in the cell, may be an attempt to compensate for the observed overall decrease in NADH and ATP levels after treatment with **1**. But counter to that, the observed downregulation of ATP synthesis genes is not in line with the cells experiencing an increased ATP demand.

### Evaluation of oxidative stress

The response of the cells to compound **1** showed some similarities to disulfide stress which has similarities to oxidative stress; however, the RNA sequencing data did not provide evidence for a triggered oxidative stress response. Therefore, we analysed whether **1** can cause the generation of Reactive Oxygen Species (ROS), using the fluorescent ROS reporter 2’, 7’‐Dichlorodihydrofluorescein diacetate (H2DCFDA). For this experiment, cells were grown in DMM which results in slower growth (doubling time of 4.5 h compared to 1 h for LB) but also a slower effect of **1** treatment which affects growth after ca. 60 min incubation (Figure S6C). The delay in effect is most likely to the slower growth rate compared to when cells grow on LB, however, we cannot exclude that other components of the media may also affect the activity of the gold compound.

**Figure 5:**
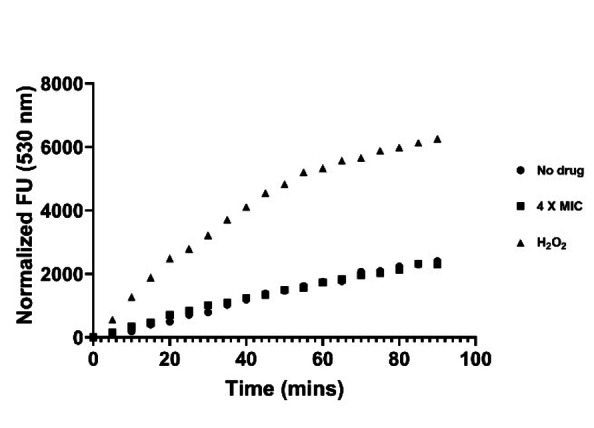
Fluorescence unit (excitation 488 nm and emission 530 nm) at each time point is normalized against the emission at T0. The graph plots the median normalized fluorescence emission value of data from 2 biological replicates with 3 technical replicates each for 90 min for 4×MIC of **1** and the controls.

ROS generation was monitored over an interval of 90 min as the effect of compound **1** is prominent in this time interval, but still not causing marked bactericidal effects. **1** treatment over a wide concentration range (between 0.0625×MIC up to 4×MIC values) did not result in any detectable ROS formation, whereas the positive control treatment, H_2_O_2_, gave clear ROS production (Figure 5 and S6A). The endpoint values at 90 minutes of the experiment are plotted in Figure S6B. These results are in line with the observed absence of upregulation of oxidative stress genes in the RNA sequencing experiment.

### Probability of occurrence of mutation

A bacterial culture is full of cells containing spontaneous mutations as a result of errors in DNA replication. To test whether such spontaneous mutations can generate resistance to **1**, a one‐step plating experiment was performed where large amounts of cells are plated on medium containing a high concentration (5×MIC) of **1**. The experiment was conducted three times; each time 2 to 3×10^7^ cells were used. No colonies appeared in each experiment, also not after prolonged incubation (up to 30 days) which would allow for the generation of mutations on the plate in cells that were not immediately killed. An image of the control and treated plates after 2 days of incubation is shown in Figure S7. Thus, the chance of resistance development due to a spontaneous mutation is less than 10^−7^. A frequency of resistance development by mutation lower than 10^−6^ is considered suitable for any potential drug candidate.

### Ex vivo toxicity studies

For any compound to be a successful drug candidate, the toxic effect of the compound on mammalian tissue has to be studied. Precision‐cut tissue slices are widely used for toxicology studies as they very closely resemble the tissue and cell composition of the organ of origin.^[39]^ In this model, all cells remain in their natural environment maintaining the original cell‐cell and cell‐matrix contacts, which are lacking in classical 2D cell cultures *in vitro*. Of note, this method is a FDA‐approved model for drug toxicity and metabolism studies, and is also useful to determine drug uptake/efflux mechanisms. Recently, we have successfully used the PCTS method to study the toxic effects of experimental anticancer organometallic compounds, including aminoferrocene‐containing prodrugs, ruthenium‐based kinase inhibitors, as well as experimental gold anticancer agents.^[40]^

**Figure 6:**
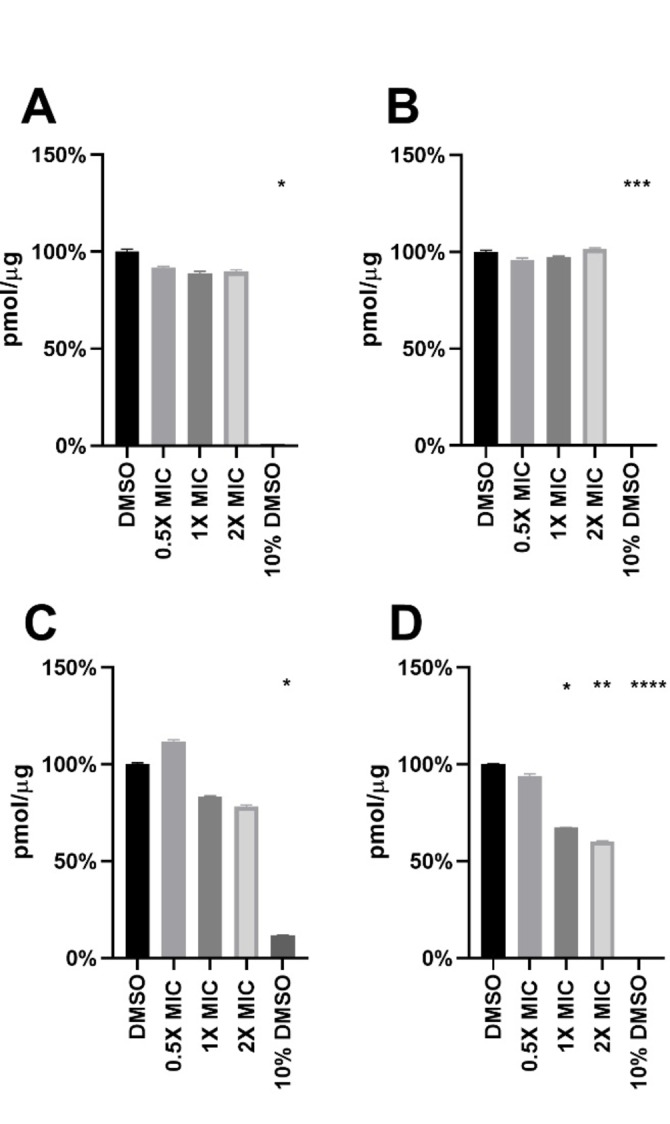
Tissue toxicity test of compound **1** on mouse precision cut tissue slices. The y‐axis shows the concentration of ATP per μg of protein. The x‐axis shows the different conditions of the study as individual columns. **A**) Liver 24 h (n=3), **B**) Liver 48 h (n=2), **C**) Kidney 24 h (n=4), **D**) Kidney 48 h (n=3). DMSO‐1.25 % as negative control (corresponding to the highest concentration used in experimental samples) 10 % DMSO was used as a positive control for the experiment; * *p*<0.05, ** *p*<0.01, *** *p*<0.001, and **** *p*<0.0001 (Kruskal‐Wallis test for A&C, ordinary one‐way Anova for B&D).

Mouse liver and kidney precision‐cut tissue slices were incubated with the compound, and the toxic effect was determined by measuring the ATP/protein level of the tissue slices after 24 and 48 hours. For the 48 hours samples, slices were re‐incubated in fresh media containing fresh compounds after 24 hours. The obtained results are plotted in Figure 6, and show that **1** did not have any significant toxic effect on mouse liver and kidney tissue compared to the DMSO control (*p*<0.05) after 24 hours. (Figure 6A, C). After 48 hours of incubation with the metallodrug, some toxic effects could be noted in kidney tissue slices (Figure 6D), while liver tissue slices were undamaged (Figure 6B). Specifically, kidney viability was reduced to ca. 60 % vs control upon treatment with compound **1** at 1×MIC after 48 h.

## Conclusions

Antibiotic resistance development in pathogens poses a significant threat to human health and urges the development of novel antibacterial agents. Here, we show that an organogold compound (**1**) featuring a bidentate C^N scaffold can be considered as a candidate antibacterial against drug resistant bacteria. The compound is a fast acting, bactericidal drug which is effective on a range of Gram‐positive bacterial strains including *B. subtilis* and *S. aureus* strains, but does not cause significant tissue toxicity *ex vivo* in mouse liver precision‐cut tissue slices. Toxic effects appear in kidney slices after only after 48 h of continuous incubation with the compound. Our studies show that in *B. subtilis* the compound rapidly decreases the energy levels of the cells, but does not have any effect on bacterial membrane permeability or membrane potential, neither on ROS production. This is different with respect to other antimicrobial gold complexes, including auranofin. A number of stress response genes and active metal transporter genes were shown to be upregulated in response to treatment with the compound. The probability of development of mutation in *B. subtilis* is less than 10^−7^, which also suggest that there is not one specific target for **1**, as the chance of development of resistant colonies by mutation would be higher if the compound had only one specific target in the cell. Overall, the mechanism of antibacterial action of the organogold compound **1** appears to be multimodal in nature, activating a number of cellular stress response pathways, but different with respect to other metal‐based complexes and disulfide stress‐inducing agents. Ongoing studies in our labs are focusing on the identification of possible bacterial protein targets for **1** via proteomic studies.

## Experimental Section

### Gold compounds

The cyclometalated Au(III) complexes with bidentate C^N ligands ‐ [Au(C^CH2^N)Cl_2_] (**1**, C^CH2^N=2‐benzylpyridine),^[41]^ [Au(C^CO^N)Cl_2_] (**2**, C^CO^N=2‐benzoylpyridine),^[42]^ [Au(C^NH^N)Cl_2_] (**3**, C^NH^N=*N‐*phenylpyridin‐2‐amine),^[43]^ [Au(bipy^dmb^‐H)GS][PF_6_] (**4**, bipy^dmb^‐H=cyclometalated 6‐(1,1‐dimethylbenzyl)‐2,2′‐bipyridine and GSH=gluthatione),^[21c]^ as well as the cationic coordination complexes [Au(pbzMe)Cl_2_]PF_6_ (**5**, pbzMe=1‐methyl‐2‐(pyridin‐2‐yl)‐benzimidazole)^[22]^ and [AubipyCl_2_]PF_6_ (**6‐7**, bipy=4,4’‐dimethoxy‐2,2’‐ bipyridine or 4,4’‐diamino‐2,2’‐bipyridine, respectively) were synthesized accordingly to previously published procedures. The compounds’ purity was assessed to be >95 %. Compounds stock solutions (10 mM) were freshly prepared in DMSO and stored in the freezer. Further dilutions for experiments were always made fresh from stock in either DMSO or medium and discarded after usage.

### Minimal inhibitory concentration and minimal bactericidal concentration assay

The minimal inhibitory concentration (MIC) of the compounds was tested against *Bacillus subtilis* 168 (from our lab strain)*, Escherichia coli* MG1655 (from Microbial Ecology lab, University of Groningen)*, Staphylococcus aureus* RN4220 (from our lab strain), Methicillin Resistant *Staphylococcus aureus* D22 (MRSA) (isolated from Denmark), *Enterococcus faecium* (from Dr. Zhou's lab, UMCG, Groningen), and *Enterococcus faecium* Van A (VRE) (from Prof. J.M van Dijl's lab, UMCG, Groningen) by broth micro‐dilution assay*. B. subtilis* and *E. coli* were cultured in LB‐Lennox medium (composition per liter: 10 g Tryptone, 5 g yeast extract, 5 g NaCl), *S. aureus* and MRSA were cultured in CAMH medium (composition per liter: 20 g NaCl, 17.5 g casein acid hydrolysate, 3 g beef extract, 1.5 g starch, 25 mg CaCl_2_, 12.5 mg MgCl_2_), and *E. faecium* strains were cultured in TSB medium (composition per liter: 17 g casein peptone (pancreatic), 5 g NaCl, 3 g soya peptone, 2.5 g glucose, 2.5 g K_2_HPO_4_) at 30 °C, 200 rpm. Exponentially growing bacteria were diluted to an OD_600_ of 0.005 in 200 μl of respective medium in a 96‐well plate (Greiner). Compounds were added as a two‐fold serial dilution series ranging from 100 μM to 0.195 μM. The plate was incubated in a microplate incubator at 30 °C with shaking (1000 rpm). OD_600_ was measured in a BioTek Powerwave 96‐well pate reader at time‐points 0 and 24 hours. The minimum inhibitory concentration was determined as the lowest concentration of compound that prevented the growth of bacteria after 24 hours. A difference of OD_600_ values between 0 and 24 hours of less than 0.1 was considered as inhibition of growth.

Two microliters from each well for **1** and **4**, starting with the lowest concentration at which bacterial growth was last seen and moving up the concentration range, was spotted on a LB agar plate without compounds and incubated at 30 °C. The minimum bactericidal concentration was determined as the lowest concentration of compound that prevented the growth of a colony on the LB plates without compound after 24 h. MBC assay was only performed on *B. subtilis cells*. Kanamycin was used as a positive control for both the assays.

### Killing dynamics

A *B. subtilis* culture at 0D_600_ of about 0.1 (6.05×10^6^ to 3.1×10^7^ cfus) was split into test tubes containing 1 mL of culture (for each time point) and treated with **1** at 12.5 μM concentration (1×MIC of the compound). Controls without compound treatment were also made for each time point. Samples were taken at 1.5, 3, 4.5 and 24 h of growth (in LB medium at 30 °C, shaking at 200 rpm), serially diluted and plated on LB plates without compound. After O/N growth, the number of viable bacteria at each time interval after drug treatment was determined by counting the number of cfu per mL culture.

To determine the activity of the drugs in real time, a *B. subtilis* culture was diluted in 200 μL of LB medium to an OD_600_ of 0.005 in a 96‐well plate and incubated in BioTek Synergy Mx 96‐well plate reader at 30 °C, 1000 rpm with reading at 20 min intervals till OD_600_ reached 0.2. Compound **1** was added in different concentrations (6.25 μM, 12.5 μM, 25 μM and 50 μM) and absorbance readings were taken at 20‐min intervals for a period of 1.5 h. A similar experiment was performed with **4** at different concentrations (3.125 μM, 6.25 μM, 12.5 μM and 25 μM). Kanamycin (MIC – 3.125 μM) and Nisin (MIC – 6 μg/mL) were used as controls in the experiment.

To determine the stability of the drug in the medium after 24 hours, LB medium was incubated with DMSO, **1** at 6.25, 12.5 and 25 μM and kept in a microplate shaker overnight at 30 °C without shaking. *Bacillus* at OD_600_ 0.005 was added to the pre‐incubated medium for MIC assays. A MIC assay in medium without pre‐incubated compound was also performed as a control.

### Membrane permeability

The effect of compound **1** on membrane permeability was measured using the live/dead BacLight Bacterial Viability kit (Invitrogen). Exponentially growing *B. subtilis* cells were incubated with DMSO (1.25 %), Nisin (6 μg/mL), **1** (12.5 μM) or without compound for 15 min (10 minutes for the Nisin sample) at 30 °C with shaking (200 rpm). Subsequently, Propidium iodide (PI) (final concentration: 30 μM) and SYTO9 (final concentration: 5 μM) were used to stain the DNA (incubated in the dark for 15 minutes). Cells were washed with PBS and concentrated 10 times, mounted on agarose pads and imaged under a microscope. Green and red fluorescence were imaged using a Nikon Ti‐E microscope (Nikon Instruments, Tokyo, Japan) with Hamamatsu OrcaFlash 4.0 camera using FITC and TriTC fluorescence filters. Microscopy images were analysed using ImageJ software and cells were counted using the chain tracer plugin. A total of around 400 cells (200 from different technical replicates for each biological replicates) were analysed for each condition.

### REMA assay

The effect of the compounds on the respiratory activity of the cell was measured by the REMA assay (Resazurine Microtiter Assay). *B. subtilis* cells at 0D_600_ 0.005 were incubated in DMM media (Davis Minimal Medium, composition per liter: 2 g casaminoacids, 7 g K_2_HPO_4_, 3 g KH_2_PO_4_, 0.1 g MgSO_4_.7H_2_O, 1 g (NH_4_)_2_SO_4_, 0.5 g tri‐sodium citrate dihydrate. Addition after autoclaving: 7 g glucose, 10 mg tryptophan) with compound **1** (12.5 μM), DMSO (1.25 %), Ciprofloxacin (0.625 μg/mL), Rifampicin (0.625 μg/mL), Tetracycline (10 μg/mL) and Vancomycin (1 μg/mL). A no‐compound control was also made. All the compounds were incubated in the medium without cells to account for the background fluorescence of the compounds. Resazurin was added at a final concentration of 0.1 mg/L. Fluorescence (excitation 530 nm, emission 560 nm) was recorded for 2 h at every 20 min interval in BioTek Synergy Mx 96‐well plate reader.

### ATP assay

The ATP assay was conducted to measure the ATP level in the cells using the BacTiter‐Glo™ Microbial Cell Viability Assay (Promega). *B. subtilis* was grown to OD_600_ 0.1 and treated with **1** at 1×MIC, DMSO and CCCP (Carbonyl cyanide *m*‐chlorophenyl hydrazine, 30 μg/mL). A no‐drug control was also included. Samples were collected after incubation at room temperature for 5, 10, 15 and 30 minutes and snap‐frozen in liquid nitrogen. 100 μL of sample was added to 100 μL of solubilized BacTiter‐Glo Reagent in a 96‐well plate and incubated at room temperature in a shaker for 5 minutes. Luminescence was measured in Tecan Infinite F‐200 Pro luminometer to determine the ATP level. An ATP standard curve in the femtomolar range was also measured with each study. Statistical analysis was done using GraphPad Prism 8. The mean of the amount of ATP for the DMSO (1.25 %) sample for each replicate at each time point was considered as 100 % and the other conditions were expressed as percentages relative to such sample. Ordinary one‐way Anova was performed on the percentage data for all the samples.

### Membrane potential assay

The change in membrane potential upon treatment with compound **1** was studied by measuring the fluorescence emitted by DiSC_3_‐5 (Molecular probes). DiSC_3_‐5 was diluted in 1×PIPES buffer (composition per 100 mL (10×): 0.58 g NaCl, 3 g PIPES in 1 M NaOH (diluted to pH 7, 0.2 g MgCl_2_.6H_2_O) and dispensed in a 96‐well microplate at a working solution of 3 μM (final volume 200 μl). Fluorescence (excitation 643 nm, emission 666 nm) was measured in a BioTek Synergy Mx 96‐well plate reader. After stabilization of the signal (15 min) 100 μl of *B. subtilis* cells at a final OD_600_ of 0.3 was added to the wells. The signal was allowed to stabilize again (5 min) followed by the addition of **1** (12.5 μM) and Nisin (1.5 μg/mL), used as positive control. No‐cell controls were included to verify the stability of the dye and also to determine whether the compounds interfered with the test. Fluorescence was measured in a BioTek Synergy Mx 96‐well plate reader for 60 min at 1 min interval after addition of the drug.

### *Ex vivo* toxicity assay

Mouse liver and kidney was sliced into precision‐cut tissue slices of 5 mg as described,^[44]^ and put in 24 well plates containing WilliamE medium substituted with glucose (1.375 g/500 mL WilliamE medium) and antibiotics (gentamycine 500 μL/500 mL WilliamE medium for liver tissues and streptomycin 5 mL/500 mL WilliamE medium for kidney tissue). The tissues were incubated with DMSO and **1** at various concentrations for 24 hours at 37 °C. The first sample was taken at time point zero. Tissue samples for the 48 hours incubation time point were transferred into plates with fresh medium and antibiotic test compounds. The tissue was homogenized in SONOP (Sonification solution – 70 % v/v Ethanol containing 2 mM EDTA, pH −10.9) and snap frozen in liquid nitrogen. The tissue was homogenized using a mini bead beater for 45 sec twice followed by centrifugation at 13000 rpm for 5 min to pellet the cell debris and protein. ATP levels in the supernatant were determined using a Roche ATP assay kit in the Synergy HT plate reader. The pellet was dried at 37 °C for 24 h to be used for protein determination. The pellet was incubated with 5 M NaOH at 37 °C water bath for 30 min, followed by homogenization using the mini bead beater for 40 sec. The protein concentration was determined using the Bio‐Rad RC Dc Protein Assay at 655 nm. Calibration curves were prepared for ATP and protein using ATP standard and BSA stock solutions respectively. The ATP/protein level in the tissues was an indication of the viability of the tissue. Statistical analysis was performed using GraphPad Prism 8. Shapiro‐Wilk normality test was performed on the data. The 24 h data was non‐parametric and the 48 h data was parametric. So, Kruskal‐Wallis‐Dunn test was performed for all the replicates for each condition using the DMSO as a control for the 24 h samples. Ordinary one‐way Anova was performed using the same conditions for the 48 h sample.

The experiments were approved by the Animal Ethical Committee of the University of Groningen (CCD number AVD105002017884) and were performed in accordance with the EU directive 2010/63/EU for animal experiments.

### RNA sequencing

*RNA extraction* ‐ Total RNA was extracted from *B. subtilis* cells treated with **1** at 0.2×MIC for 45 min and 60 min following a standard Trizol RNA extraction protocol^[45]^ with modifications. The initial lysozyme incubation of the cells was replaced with disruption of the cells using Thermo Savant Fast Prep FP120 disruptor AT 6 m/sec for 40 sec. DMSO treated cells were included as controls for the same time points. Total RNA was sequenced by Illumina sequencing (Macrogen, Europe) to generate 20 M paired reads of read length 100bp PE.

*RNA sequencing data analysis* – FastQ files were run through a BowTie2 – Top Hat‐ Sam Tool pipeline to generate the BAM files. Further analysis of the BAM files was performed using T‐REx2: Comprehensive Gene Expression Data Analysis^[35]^ and Subtiwiki.^[36]^ Raw counts for the experiments are included as Supporting Information.

### ROS measurement assay

The effect of the compound on the generation of oxidative stress in the cell was studied using the fluorescent dye 2’,7’‐Dichlorodihydrofluorescein diacetate (H2DCFDA). Cells were grown in DMM media and incubated with the dye (5 μM) in the dark for 1 h, washed with fresh media, followed by addition of **1** in two‐fold serial dilution from 50 μM to 0.78 μM. H_2_O_2_ was used as a positive control and a no drug sample was used as a negative control. Fluorescence (excitation – 488 nm, emission – 530 nm) and absorbance (600 nm, to monitor growth) were measured using the BioTek Synergy Mx 96‐well plate reader for 12 h at 5 min interval. The data at each time point was normalized against the T0 value, and fluorescence was plotted for 90 min following treatment with the compound. One‐way Anova was performed for all the replicates data keeping the ‘No drug’ as the control set. All statistical analysis was performed using GraphPad Prism 8.

### Probability of occurrence of mutation

A fresh bacterial culture was plated on LB agar plates containing 5×MIC **1**. The same culture was also plated on a LB agar plate without any antibiotics to determine the number of bacteria in the culture. The frequency of occurrence of mutation was defined as the number of colonies appearing on the compound‐containing plate relative to the total number of cells plated as determined by counting colonies from the untreated culture after 24 h. The compound‐containing plates were further incubated for a period of 30 days with frequent inspection for the appearance of resistant colonies.

## Conflict of interest

The authors declare no conflict of interest.

## Supporting information

As a service to our authors and readers, this journal provides supporting information supplied by the authors. Such materials are peer reviewed and may be re‐organized for online delivery, but are not copy‐edited or typeset. Technical support issues arising from supporting information (other than missing files) should be addressed to the authors.

Supporting Information

## References

[1] G. J. Higby , Gold Bull. 1982, 15, 130–140. 11614517 10.1007/BF03214618

[2a] A. Casini , in Metallo-Drugs: Development and Action of Anticancer Agents, Vol. 18 (Eds.: A. Sigel , H. Sigel , E. Freisinger , R. K. O. Sigel ), Walter de Gruyter GmbH, 2018, pp. 199–217;

[2b] S. Nobili , E. Mini , I. Landini , C. Gabbiani , A. Casini , L. Messori , Med. Res. Rev. 2010, 30, 550–580. 19634148 10.1002/med.20168

[3] R. Koch , Dtsch. Med. Wochenschr. 1890, 16, 756–757.

[4] R. Aminov , Front. Microbiol. 2010, 1, 1–7. 21687759 10.3389/fmicb.2010.00134 PMC3109405

[5] S. Sengupta , M. Chattopadhyay , H.-P. Grossart , Front. Microbiol. 2013, 4, 1–13. 23487476 10.3389/fmicb.2013.00047 PMC3594987

[6] A. Frei , Antibiotics 2020, 9, 90.

[7] B. Đ. Glišić , M. I. Djuran , Dalton Trans. 2014, 43, 5950–5969. 24598838 10.1039/c4dt00022f

[8] I. Ozdemir , N. Temelli , S. Günal , S. Demir , Molecules 2010, 15, 2203–2210. 20428038 10.3390/molecules15042203 PMC6257235

[9a] İ. Özdemir , A. Denizci , H. T. Öztürk , B. Çetinkaya , Appl. Organomet. Chem. 2004, 18, 318–322;

[9b] A. Vellé , R. Maguire , K. Kavanagh , P. J. Sanz Miguel , D. Montagner , ChemMedChem 2017, 12, 841–844; 28463422 10.1002/cmdc.201700257

[9c] J. R. Stenger-Smith , P. K. Mascharak , ChemMedChem 2020, 15, 2136–2145. 33025735 10.1002/cmdc.202000608

[10a] G. Roymahapatra , S. M. Mandal , W. F. Porto , T. Samanta , S. Giri , J. Dinda , O. L. Franco , P. K. Chattaraj , Curr. Med. Chem. 2012, 19, 4184–4193; 22680631 10.2174/092986712802430090

[10b] T. Samanta , G. Roymahapatra , W. F. Porto , S. Seth , S. Ghorai , S. Saha , J. Sengupta , O. L. Franco , J. Dinda , S. M. Mandal , PLoS One 2013, 8, e58346. 23554886 10.1371/journal.pone.0058346 PMC3598898

[11] D. G. Brown , T. L. May-Dracka , M. M. Gagnon , R. Tommasi , J. Med. Chem. 2014, 57, 10144–10161. 25402200 10.1021/jm501552x

[12] M. B. Harbut , C. Vilchèze , X. Luo , M. E. Hensler , H. Guo , B. Yang , A. K. Chatterjee , V. Nizet , W. R. Jacobs , P. G. Schultz , F. Wang , Proc. Natl. Acad. Sci. USA 2015, 112, 4453. 25831516 10.1073/pnas.1504022112 PMC4394260

[13] J. P. Owings , N. N. McNair , Y. F. Mui , T. N. Gustafsson , A. Holmgren , M. Contel , J. B. Goldberg , J. R. Mead , FEMS Microbiol. Lett. 2016, 363. 10.1093/femsle/fnw148 27279627

[14] C. Schmidt , B. Karge , R. Misgeld , A. Prokop , R. Franke , M. Brönstrup , I. Ott , Chem. Eur. J. 2017, 23, 1869–1880. 27865002 10.1002/chem.201604512

[15] A. Holmgren , J. Lu , Biochem. Biophys. Res. Commun. 2010, 396, 120–124. 20494123 10.1016/j.bbrc.2010.03.083

[16] R. C. Fahey , W. C. Brown , W. B. Adams , M. B. Worsham , J. Bacteriol. 1978, 133, 1126–1129. 417060 10.1128/jb.133.3.1126-1129.1978 PMC222142

[17] J. J. Criado , J. A. Lopez-Arias , B. Macias , L. R. Fernandez-Lago , J. M. Salas , Inorg. Chim. Acta 1992, 193, 229–235.

[18a] R. V. Parish , J. Mack , L. Hargreaves , J. P. Wright , R. G. Buckley , A. M. Elsome , S. P. Fricker , B. R. C. Theobald , Dalton Trans. 1996, 69–74;

[18b] R. V. Parish , Met.-Based Drugs 1999, 6, 271–276. 18475902 10.1155/MBD.1999.271 PMC2365175

[19] A. Pintus , M. C. Aragoni , M. A. Cinellu , L. Maiore , F. Isaia , V. Lippolis , G. Orrù , E. Tuveri , A. Zucca , M. Arca , J. Inorg. Biochem. 2017, 170, 188–194. 28260677 10.1016/j.jinorgbio.2017.02.015

[20] R. Gupta , C. Rodrigues Felix , M. P. Akerman , K. J. Akerman , C. A. Slabber , W. Wang , J. Adams , L. N. Shaw , Y.-C. Tse-Dinh , O. Q. Munro , K. H. Rohde , Antimicrob. Agents Chemother. 2018, 62, e01696–01617. 29483110 10.1128/AAC.01696-17 PMC5923144

[21a] A. Casini , M. C. Diawara , R. Scopelliti , S. M. Zakeeruddin , M. Grätzel , P. J. Dyson , Dalton Trans. 2010, 39, 2239–2245; 20162197 10.1039/b921019a

[21b] B. Bertrand , S. Spreckelmeyer , E. Bodio , F. Cocco , M. Picquet , P. Richard , P. Le Gendre , C. Orvig , M. A. Cinellu , A. Casini , Dalton Trans. 2015, 44, 11911–11918; 26060937 10.1039/c5dt01023c

[21c] S. Carboni , A. Zucca , S. Stoccoro , L. Maiore , M. Arca , F. Ortu , C. Artner , B. K. Keppler , S. M. Meier-Menches , A. Casini , M. A. Cinellu , Inorg. Chem. 2018, 57, 14852–14865; 30457328 10.1021/acs.inorgchem.8b02604

[21d] B. Aikman , M. N. Wenzel , A. F. Mósca , A. De Almeida , W. T. Klooster , S. J. Coles , G. Soveral , A. Casini , Inorganics 2018, 6, 123.

[22] A. de Almeida , A. F. Mósca , D. Wragg , M. Wenzel , P. Kavanagh , G. Barone , S. Leoni , G. Soveral , A. Casini , Chem. Commun. (Camb.) 2017, 53, 3830–3833. 28304043 10.1039/c7cc00318h

[23] A. Casini , A. Guerri , C. Gabbiani , L. Messori , J. Inorg. Biochem. 2008, 102, 995–1006. 18289690 10.1016/j.jinorgbio.2007.12.022

[24] V. Graziani , A. Marrone , N. Re , C. Coletti , J. A. Platts , A. Casini , Chem. Eur. J. 2017, 23, 13802–13813. 28776779 10.1002/chem.201703092

[25] L. Mazzei , M. N. Wenzel , M. Cianci , M. Palombo , A. Casini , S. Ciurli , ACS Med. Chem. Lett. 2019, 10, 564–570. 30996797 10.1021/acsmedchemlett.8b00585 PMC6466819

[26] S. R. Thomas , A. Casini , Curr. Opin. Chem. Biol. 2020, 55, 103–110. 32086166 10.1016/j.cbpa.2019.12.007

[27a] M. N. Wenzel , R. Bonsignore , S. R. Thomas , D. Bourissou , G. Barone , A. Casini , Chem. Eur. J. 2019, 25, 7628–7634; 30990916 10.1002/chem.201901535 PMC6594228

[27b] S. R. Thomas , R. Bonsignore , J. Sanchez Escudero , S. M. Meier-Menches , C. M. Brown , M. O. Wolf , G. Barone , L. Y. P. Luk , A. Casini , ChemBioChem 2020, 21, 3071–3076. 32511840 10.1002/cbic.202000262 PMC7689846

[28] A. de Almeida , B. L. Oliveira , J. D. G. Correia , G. Soveral , A. Casini , Coord. Chem. Rev. 2013, 257, 2689–2704.

[29] G. Nahler, *Dictionary of Pharmaceutical Medicine*, Springer, Vienna, **2009**.

[30] K. Minori , L. B. Rosa , R. Bonsignore , A. Casini , D. C. Miguel , ChemMedChem 2020, 15, 2146–2150. < 32830445 10.1002/cmdc.202000536 PMC7756297

[31] K. S. Gurusamy , R. Koti , C. D. Toon , P. Wilson , B. R. Davidson , Cochrane Database Syst. Rev. 2013, 11, Cd010427. 10.1002/14651858.CD010427.pub2 PMC11299151 24242704

[32] G. N. Moll , G. C. K. Roberts , W. N. Konings , A. J. M. Driessen , Antonie van Leeuwenhoek 1996, 69, 185–191. 8775978 10.1007/BF00399423

[33] S. Zhang , J. Huang , Z. Zhao , Y. Cao , B. Li , Front Public Health. 2020, 8, 574968. 33194975 10.3389/fpubh.2020.574968 PMC7655780

[34] M. B. Tol , D. Morales Angeles , D.-J. Scheffers , Antimicrob. Agents Chemother. 2015, 59, 3683–3686. 25870072 10.1128/AAC.04781-14 PMC4432147

[35] A. de Jong , S. van der Meulen , O. P. Kuipers , J. Kok , BMC Genomics 2015, 16, 663. 26335208 10.1186/s12864-015-1834-4 PMC4558784

[36a] B. Zhu , J. Stülke , Nucleic Acids Res. 2017, 46, D743–D748; 10.1093/nar/gkx908 PMC5753275 29788229

[36b] U. Mäder , A. G. Schmeisky , L. A. Flórez , J. Stülke , Nucleic Acids Res. 2011, 40, D1278–D1287. 22096228 10.1093/nar/gkr923 PMC3245094

[37] P. Nicolas , U. Mäder , E. Dervyn , T. Rochat , A. Leduc , N. Pigeonneau , E. Bidnenko , E. Marchadier , M. Hoebeke , S. Aymerich , D. Becher , P. Bisicchia , E. Botella , O. Delumeau , G. Doherty , E. L. Denham , M. J. Fogg , V. Fromion , A. Goelzer , A. Hansen , E. Härtig , C. R. Harwood , G. Homuth , H. Jarmer , M. Jules , E. Klipp , L. Le Chat , F. Lecointe , P. Lewis , W. Liebermeister , A. March , R. A. T. Mars , P. Nannapaneni , D. Noone , S. Pohl , B. Rinn , F. Rügheimer , P. K. Sappa , F. Samson , M. Schaffer , B. Schwikowski , L. Steil , J. Stülke , T. Wiegert , K. M. Devine , A. J. Wilkinson , J. Maarten van Dijl , M. Hecker , U. Völker , P. Bessières , P. Noirot , Science 2012, 335, 1103. 22383849 10.1126/science.1206848

[38] L. I. O. Leichert , C. Scharf , M. Hecker , J. Bacteriol. 2003, 185, 1967–1975. 12618461 10.1128/JB.185.6.1967-1975.2003 PMC150141

[39a] I. A. de Graaf , G. M. Groothuis , P. Olinga , Expert Opin. Drug Metab. Toxicol. 2007, 3, 879–898; 18028031 10.1517/17425255.3.6.879

[39b] A. R. Parrish , A. J. Gandolfi , K. Brendel , Life Sci. 1995, 57, 1887–1901. 7475939 10.1016/0024-3205(95)02176-j

[40a] B. Bertrand , L. Stefan , M. Pirrotta , D. Monchaud , E. Bodio , P. Richard , P. Le Gendre , E. Warmerdam , M. H. de Jager , G. M. M. Groothuis , M. Picquet , A. Casini , Inorg. Chem. 2014, 53, 2296–2303; 24499428 10.1021/ic403011h

[40b] S. Daum , V. F. Chekhun , I. N. Todor , N. Y. Lukianova , Y. V. Shvets , L. Sellner , K. Putzker , J. Lewis , T. Zenz , I. A. de Graaf , G. M. Groothuis , A. Casini , O. Zozulia , F. Hampel , A. Mokhir , J. Med. Chem. 2015, 58, 2015–2024; 25633601 10.1021/jm5019548

[40c] S. Spreckelmeyer , N. Estrada-Ortiz , G. G. H. Prins , M. van der Zee , B. Gammelgaard , S. Sturup , I. A. M. de Graaf , G. M. M. Groothuis , A. Casini , Metallomics 2017, 9, 1786–1795; 29104982 10.1039/c7mt00271h

[40d] N. Estrada-Ortiz , F. Guarra , I. A. M. de Graaf , L. Marchetti , M. H. de Jager , G. M. M. Groothuis , C. Gabbiani , A. Casini , ChemMedChem 2017, 12, 1429–1435. 28741878 10.1002/cmdc.201700316

[41] M. A. Cinellu , A. Zucca , S. Stoccoro , G. Minghetti , M. Manassero , M. Sansoni , Dalton Trans. 1995, 2865–2872.

[42] Y. Fuchita , H. Ieda , A. Kayama , J. Kinoshita-Nagaoka , H. Kawano , S. Kameda , M. Mikuriya , Dalton Trans. 1998, 4095–4100.

[43] M. A. Cinellu , A. Zucca , S. Stoccoro , G. Minghetti , M. Manassero , M. Sansoni , Dalton Trans. 1996, 4217–4225.

[44] I. A. de Graaf , P. Olinga , M. H. de Jager , M. T. Merema , R. de Kanter , E. G. van de Kerkhof , G. M. Groothuis , Nat. Protoc. 2010, 5, 1540–1551. 20725069 10.1038/nprot.2010.111

[45] E. Villa-Rodríguez , C. Ibarra-Gámez , S. de Los Santos-Villalobos , J. Microbiol. Methods 2018, 147, 14–16. 29474841 10.1016/j.mimet.2018.02.011

