# Supplementary material for: An Organogold Compound as Potential Antimicrobial Agent against Drug‐Resistant Bacteria: Initial Mechanistic Insights
Source: ChemMedChem. 2021 Jul 23;16(19):3060–70. doi: 10.1002/cmdc.202100342 (PMC8518660; doi:10.1002/cmdc.202100342)
Supplement: Supplementary file 1 — Supporting Information [file CMDC-16-3060-s001.pdf]

ChemMedChem

Supporting Information

An Organogold Compound as Potential Antimicrobial Agent against Drug-Resistant Bacteria: Initial Mechanistic Insights

Parichita Chakraborty, Dorenda Oosterhuis, Riccardo Bonsignore, Angela Casini, Peter Olinga, and Dirk-Jan Scheffers*

Author Contributions

P.C. Conceptualization:Equal; Data curation:Lead; Formal analysis:Lead; Investigation:Lead; Methodology:Equal; Visualization:Lead; Writing – original draft:Lead; Writing – review & editing:Lead
D.O. Methodology:Supporting; Resources:Supporting
R.B. Resources:Lead; Visualization:Supporting; Writing – review & editing:Supporting
A.C. Conceptualization:Supporting; Resources:Lead; Supervision:Supporting; Writing – original draft:Supporting; Writing – review & editing:Equal
P.O. Conceptualization:Equal; Methodology:Supporting; Project administration:Supporting; Supervision:Equal; Writing – original draft:Equal; Writing – review & editing:Supporting
D.-J.S. Conceptualization:Equal; Project administration:Lead; Supervision:Equal; Writing – original draft:Lead; Writing – review & editing:Lead

Supplementary Information Available

Structure	Name	*B. subtilis*	*E. coli*
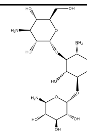	Kanamycin	1.56-3.12	12.5-25
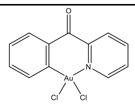	**2**	50-100	>100
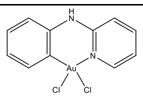	**3**	>100	>100
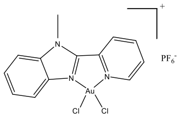	**5**	50-100	50-100
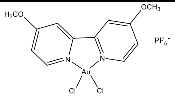	**6**	50-100	>100
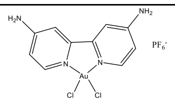	**7**	>100	50-100

Table S1: MIC values (μm) of the different gold compounds in *B. subtilis* and *E. coli.* Kanamycin is shown here as a control. MIC value is reported as a range between the highest concentration allowing bacterial growth and lowest concentration inhibiting bacterial growth after 24 hours. Values shown here are the mean values performed for 2 biological replicates with 2 technical replicates each in case of *B. subtilis* and *E. coli.*

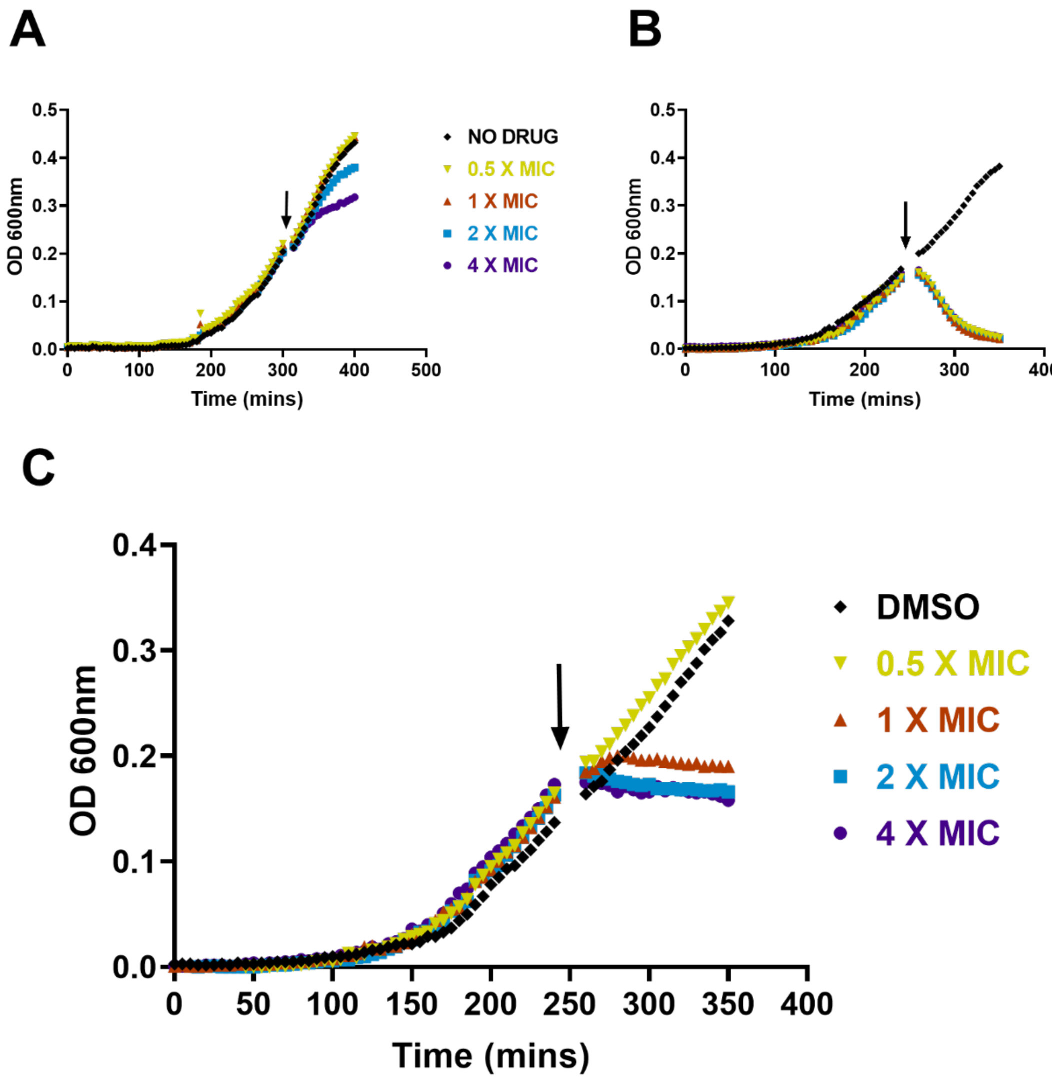

Figure S1: Killing dynamic curve of **A**: Kanamycin, **B**: Nisin, **C**: compound **4**. The y-axis shows the absorbance at 600 nm. The x-axis shows the time in minutes. The arrow indicates the point of drug entry in each experiment. The graph represents the median value of three technical replicates at each time point.

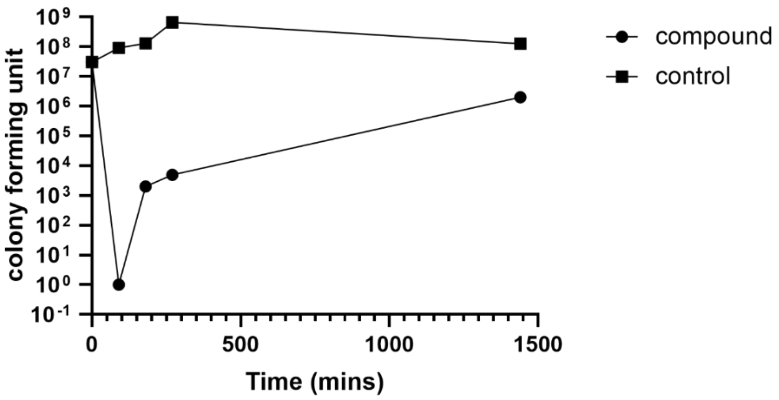

Figure S2: Graph showing the colony forming units in treated and non-treated samples at different time points. The x-axis represents the time in minutes and the y- axis represents the cfu. The cfu is calculated as (Number of colonies X Dilution factor)/ml. The experiment was performed with three biological replicates with similar results.

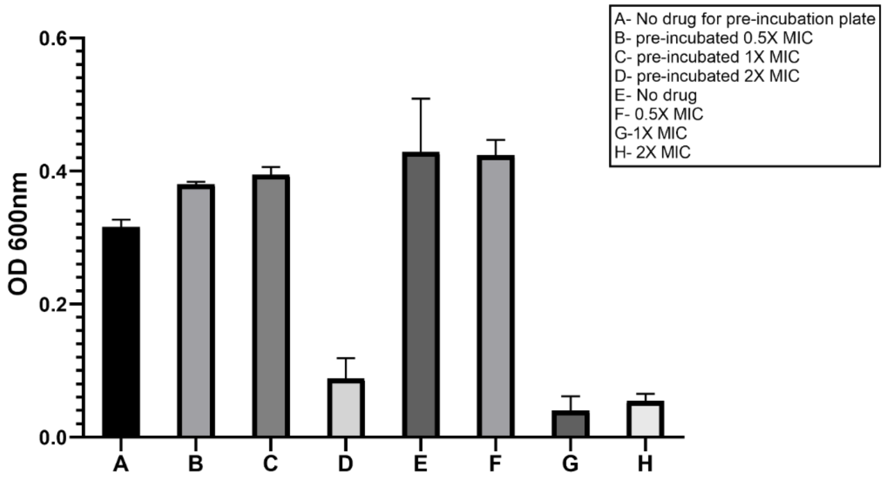

Figure S3: Stability of compound **1** after 24 hours' incubation in medium. The y-axis shows the OD at 600 nm. The x-axis represents the different conditions of study as individual columns. The columns represent the mean with SEM for 3 technical replicates of 1 biological replicate. The experiment was repeated twice with similar results.

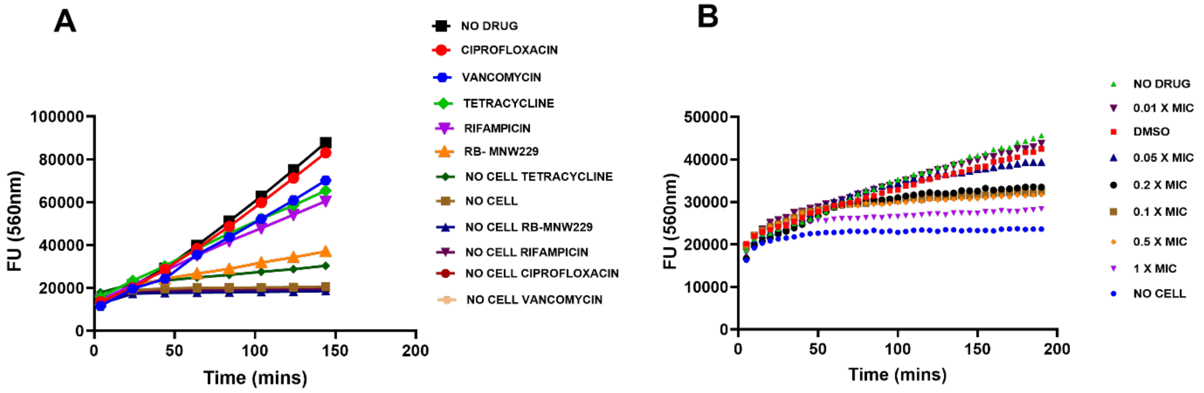

Figure S4: **A**- Effect of different known antibiotics on the NADH+H$^+$ level of *B. subtilis.* The y-axis shows the fluorescent values at 530 nm excitation and 560 nm emission. The x-axis shows the time in minutes. The data points represent the mean values of three technical replicates for one biological replicate for each time point. The experiment was performed with three biological replicates with similar result. **B**- Effect of different concentrations of **1** on the NADH+H$^+$ level of *B. subtilis.* The data points represent the median value of three technical replicates for one biological replicate for each time point. The experiment was performed with two biological replicates with similar result.

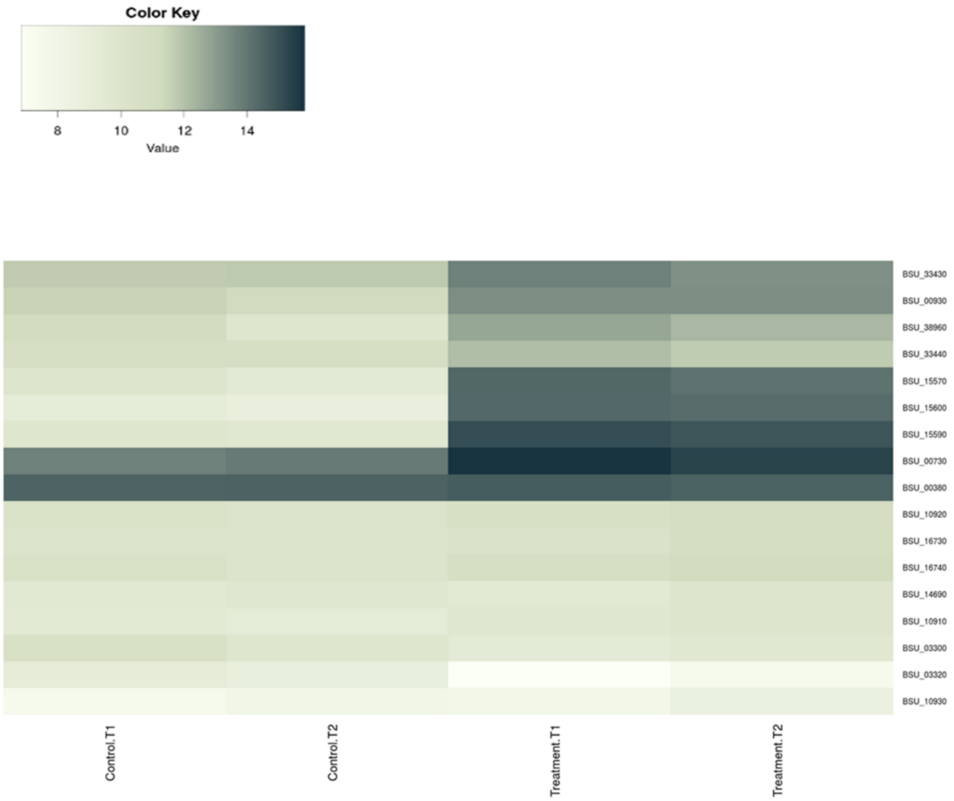

Figure S5: Heat map of expression level of metal metabolism genes in control and compound **1**-treated samples after 45 min (T1) and 60 min (T2). Each row represents one gene and each column represents the result analyzed from two biological samples of one condition.

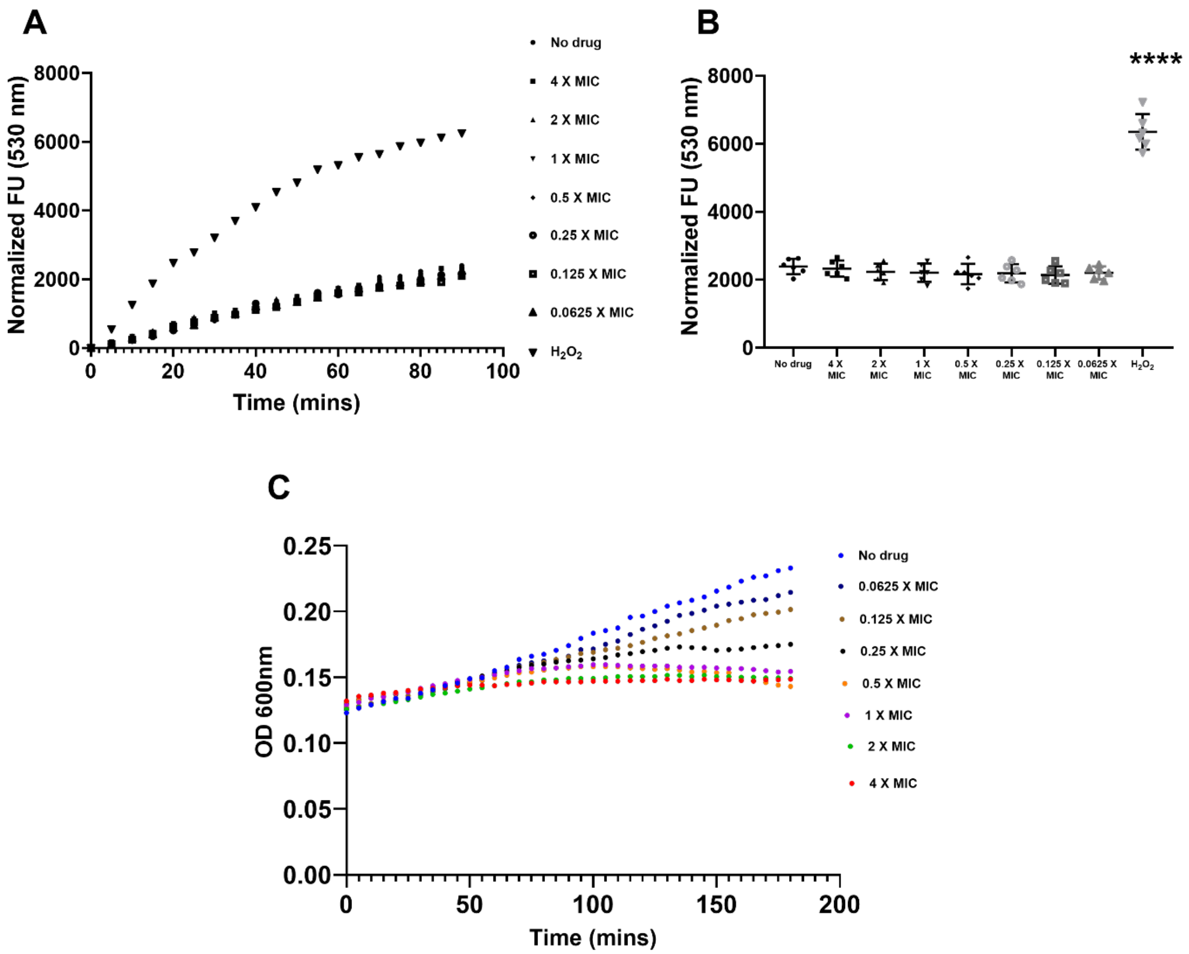

Figure S6: Fluorescence unit (excitation-488 nm and emission- 530 nm) at each time point is normalized against the emission at T0. **A**- The normalized fluorescence emission for 90 min for each condition. **B**- The normalized fluorescence emission for all the replicates for each condition recorded at the 90th minute. **** shows the significant data set at $p<0.0001$ (Ordinary one way Anova). **C-** The killing dynamics curve for the different concentrations of **1** in DMM showing that the activity starts after 60 min for the highest concentration. Graph A plots the median value of data from 2 biological replicates with 3 technical replicates each, B plots the individual values of the six replicates with the mean as the central line with SD and C plots the median OD_{600} values from 2 biological replicates and 3 technical replicates.

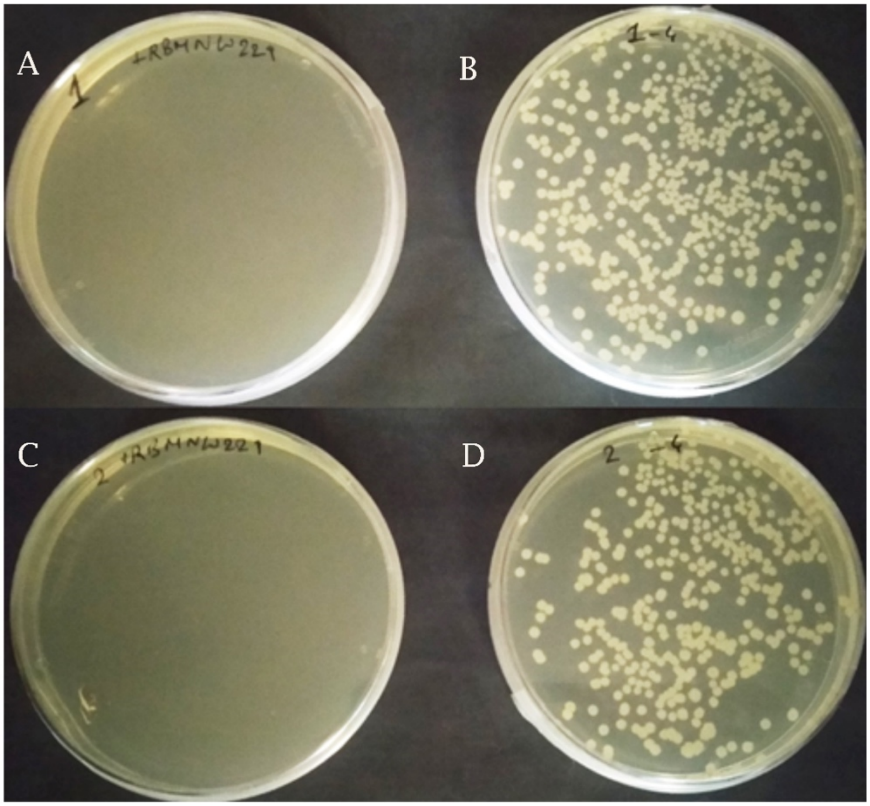

Figure S7: Images of *B. subtilis* plated on LB agar with 5X MIC (62.5μM) RB-MNW229 (**A**&**C**) and without antibiotics (**B**&**D**) after 2 days of plating.
